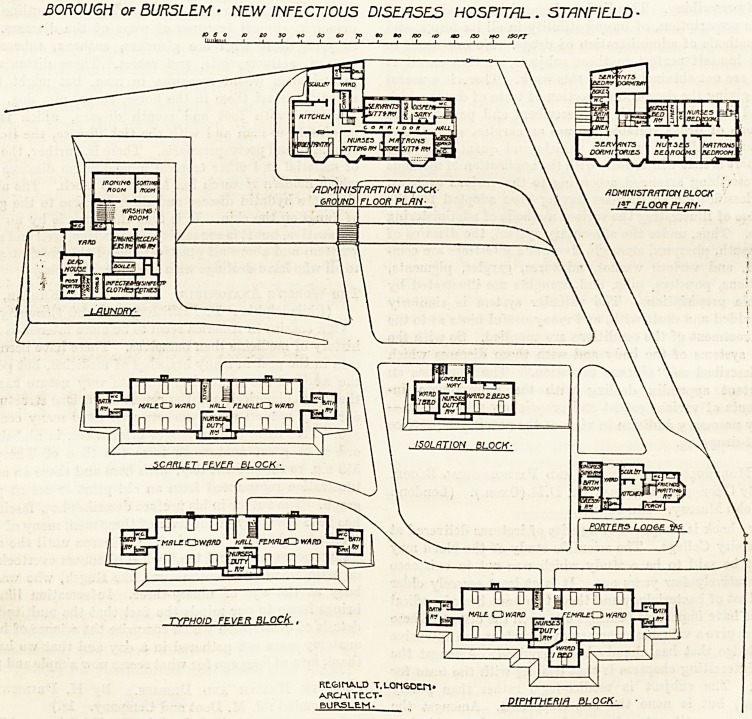# Isolation Hospital for Burslem

**Published:** 1906-09-08

**Authors:** 


### HOSPITAL ADMINISTRATION.

### CONSTRUCTION AND ECONOMICS.

### ISOLATION HOSPITAL FOR BURSLEM.

This hospital was opened in March 1906, the opening ceremony having been performed by the Mayoress, Mrs. Dobson. Previous to this year the infectious cases from Burslem had been sent to the Bradwell Joint Hospital, but the available accommodation there was becoming insufficient to meet the calls on it. It was proposed to make additions to the Bradwell Hospital; but, as these would have been of a very expensive kind, the Burslem authorities wisely decided to erect an institution of their own. The site is at Stanfield, about one mile from the Burslem Town Hall. It is nearly eight acres in extent. The entrance is to the south and is approached by way of Hamil Road and Lorne Street.

BOROUGH OF BURSLEM · NEW INFECTIOUS DISEASES HOSPITAL. STANFIELD·

The hospital consists of seven separate blocks. The porter's lodge has incorporated with it a waiting-room for patients' friends, and a bath-room and dressing-rooms for those patients who are about to be discharged. On the left of the lodge on entering the grounds is the diphtheria block for thirteen patients. There are two six-bedded wards and a single-bedded room, the latter projecting from the block in such a manner that efficient cross-ventilation could easily be obtained by windows on its north and south aspects. The sanitary blocks are placed at the ends of the wards, and each contains bath-room, closet, and sink, all properly cut off by cross-ventilated passages.

The typhoid and scarlet fever blocks are similar in size and in design, save that they are not provided with single-bedded wards. In all cases nurses' duty-rooms are placed between the wards. These have inspection windows overlooking the wards, and they are fitted up with everything necessary.

There is another small pavilion containing two wards, one having two beds and the other one bed. This is intended for the observation of doubtful cases; that is, of cases in whom the nature of the febrile symptoms cannot be determined on admission.

The laundry block is conveniently arranged for easy and economical management. It contains receiving-room, sorting-room, infected clothes-room, disinfected clothes-room, ironing-room, etc.; and this block has attached to it the mortuary and post-mortem room; but, of course, these have a separate entrance and an enclosed court.

The administrative block has incorporated with it an old villa which went with the estate, and the building seems to have been very cleverly altered and added to. The ground floor is given up to sitting-rooms for the matron, nurses, and domestic servants. There are also dispensary, cloak-room, and a good kitchen.

Returning to the blocks which constitute the hospital proper, we note that each of the large wards is 36 feet long, 26 feet wide, and 13 feet high. Each bed has therefore a wall space of 12 feet, a superficial area of 156 feet and a cubic air space of 2,028 feet. The walls are covered with adamant. All angles have been rounded off to form coves, and at the junction of the wall and the floor these coves are made of tiles. The floors are of dry pitch pine.

The windows are on the double-hung sash principle with hoppers over, and we believe that this form of window has proved quite satisfactory in other hospitals, and it has the advantage of being easier to manage than windows of the Guy's Hospital type.

Ventilators have been fixed in the external walls, and each bed has one of these ventilators opening under it and another at several feet above the bed. Extractors on the air-pump system are fixed on the roof. Doulton's double stoves are used for warming the wards.

The architectural effect of the buildings has been obtained by simplicity and proportion, and the result is said to be satisfactory. The elevations are in red brick relieved by broad bands of rough-cast. The roofs are covered with Stanley's red-brindled tiles.

The drainage has been carefully seen to, and each block is cut off from the others by intercepting chambers and traps. The sewage is treated on the "bacteria" system.

The possibility of an outbreak of fire has not been overlooked, and a four-inch main is laid on to several points of the buildings; while hand fire-extinguishing machines and buckets are supplied to the wards.

The whole of the hospital and its various adjuncts and arrangements have evidently been carefully studied; but once more we have to express our regret at the too slavish copying of Local Government Board models. It has not yet been shown that these models are the best possible in design. Why, then, will not architects try to improve on them?

The total cost of the Burslem Hospital buildings was £9,500. This sum included machinery and fittings of various kinds, and as there are forty beds in the hospital the cost per bed was £237 10s. It is certain that the site is large enough for blocks to accommodate thirty additional patients; and it is further pointed out by the architect that the administrative department has been made sufficient for seventy beds, so that when built the cost per bed of the seventy beds would be only £170. This is by no means excessive. In fact, it is low for such hospitals to cost at the present day; but we are not sure that we should like to accept the opinion that it is "a much lower cost than has been the case in recent years."

The architect was Mr. Longden, of Burslem, and the contractors were Messrs. Broadhurst and Sons, of Burslem.

## Figures and Tables

**Figure f1:**